# Converting Visitors of Physicians’ Personal Websites to Customers in Online Health Communities: Longitudinal Study

**DOI:** 10.2196/20623

**Published:** 2020-08-26

**Authors:** Qin Chen, Xiangbin Yan, Tingting Zhang

**Affiliations:** 1 School of Economics and Management University of Science and Technology Beijing Beijing China

**Keywords:** online health communities, conversion rate, multisource information, physician-generated information, patient-generated information, system-generated information, usage time

## Abstract

**Background:**

With the dramatic development of Web 2.0, increasing numbers of patients and physicians are actively involved in online health communities. Despite extensive research on online health communities, the conversion rate from visitor to customer and its driving factors have not been discussed.

**Objective:**

The aim of this study was to analyze the conversion rate of online health communities and to explore the effects of multisource online health community information, including physician-generated information, patient-generated information, and system-generated information.

**Methods:**

An empirical study was conducted to examine the effects of physician-generated, patient-generated, and system-generated information on the conversion rate of physicians’ personal websites by analyzing short panel data from 2112 physicians over five time periods in a Chinese online health community.

**Results:**

Multisource online health community information (ie, physician-generated, patient-generated, and system-generated information) positively affected the conversion rate. Physician-generated and patient-generated information showed a substitute relationship rather than a complementary relationship. In addition, the usage time of a personal website positively moderated patient-generated information, but negatively moderated physician-generated information.

**Conclusions:**

This study contributes to the electronic health literature by investigating the conversion rate of online health communities and the effect of multisource online health community information. This study also contributes to understanding the drivers of conversion rate on service websites, which can help to successfully improve the efficiency of online health communities.

## Introduction

### Background

Medical resources are both limited and irrationally distributed [1]. Large cities have the most medical services, causing many patients in small cities and countries to struggle for basic health care [2]. With the support of Health 2.0 technologies, an increasing number of internet users have realized that the internet is useful for acquiring knowledge and information on diseases and treatments [3], which has stimulated the development of online health communities (OHCs). OHCs can be divided into online physician-patient communities and online patient-patient communities according to the user composition and the communication model [4]. In this study, we focused on an online physician-patient community (hereafter referred to generally as OHC). An OHC is a platform on which patients are now able to consult physicians on health issues and the treatment of their illness anytime and anywhere. Current evidence shows that OHCs have considerably significant effects in terms of reducing medical costs, improving operational efficiency and effectiveness, enhancing the equity of medical resources, and meeting patient satisfaction [5].

Customers increasingly prefer the internet to purchase products and services, and a digital channel has become an important platform for sellers of different sectors [6]. Numerous technologies and strategies are utilized to increase website traffic; however, an increase in website traffic alone does not guarantee an increase in sales. It is also important to convert visitors into customers [7,8]. Despite the importance of the conversion rate, research on understanding and analyzing the conversion rates of service websites is limited, especially in OHCs. Service has three well-documented features: intangibility, heterogeneity, and inseparability [9]. First, most services cannot be counted, measured, inventoried, tested, and verified in advance of the sale to assure quality. Second, performance often varies from producer to producer, from customer to customer, and from day to day. Third, production and consumption of services are inseparable. Unlike the conversion rate of other types of websites, the conversion rate for a service website represents a proportion of customers to total visitors who successfully locate information [10]. In the process of choosing a physician in an OHC, the patient decides whether or not to consult online or offline after visiting a physician’s personal website [11,12], which converts the visitor of a personal website to a customer. Therefore, the conversion rate of an OHC can be defined as the proportion of customers to visitors who successfully locate information on physicians’ personal websites and decide to consult. Therefore, how to convert visitors of a personal website to customers is an essential issue for physicians and the manager of the OHC.

Customers rely on several different sources to gather information on a variety of products or services prior to purchase [13-15]. Similarly, patients rely on OHC information from multiple sources to select a physician, and the physician’s personal website gathers this OHC information (eg, online and offline information [4,5], individual and organizational information [16], and system-generated and patient-generated information [12]). Among them, system-generated information is created by the platform where physicians work, and reveals the service provider’s contribution, grade, popularity, and other characteristics. This information is independent of physicians and patients in OHCs [12]. Patient-generated information is generated by patients who have experienced medical consultation (eg, reviews and ratings), which is typically used as a clue to reflect the outcomes of the physician’s service [17-19]. System-generated information and patient-generated information have been shown to be relevant to patients’ choices [12]. However, it is unclear whether this information will also affect the conversion rate of physicians’ personal websites.

In addition, the information presented in a personal website also includes information generated by the physician’s own online behaviors and activities (ie, physician-generated information) [18]. For example, the number of articles published by a physician can be used to measure their activeness [18], knowledge contribution [5], and professional capital [20]. In addition, physicians’ online behaviors and activities are important cues to reflect their delivery process quality [12]. Patients tend to judge a physician’s service quality from two dimensions: the outcomes and delivery process of the service [12], which correspond to the definitions of patient-generated information and physician-generated information that we applied in this study. Therefore, determining whether physician-generated information is related to the conversion rate of a personal website and its relationship with patient-generated information are worth studying.

System-generated information is automatically calculated by the system using an algorithm [12] and is updated at intervals, whereas patient-generated information and physician-generated information are recorded in the physician’s personal website as of its launch. Thus, the amount of patient-generated and physician-generated information is related to the physician’s usage time of the personal website. Moreover, the longer the physician uses the website, the more familiar they become with online medical services. Similar to patients who prefer experienced physicians in traditional medical services, patients may prefer to choose a physician who is more familiar with online medical services. Thus, a physician’s usage time may affect the relationship between patient-generated information, physician-generated information, and the conversion rate of the personal website.

The main objective of this study was to investigate the impact of multisource OHC information (physician-generated information, patient-generated information, and system-generated information) on the conversion rate of physicians’ personal websites, using data from a Chinese OHC. The main research questions were: (1) Could physician-generated information, patient-generated information, and system-generated information affect the conversion rate of physicians’ personal websites in OHCs? (2) Could physician-generated and patient-generated information be used to complement the conversion rate of physicians’ personal websites in OHCs? and (3) Does the usage time of a personal website moderate the effects of physician-generated and patient-generated information on the conversion rate? If so, to what degree (strengthen or weaken)?

To answer these three research questions, we developed a research model, which was tested using data collected from 2112 physicians’ personal websites over five time periods from the Chinese OHC Haodf.com [21].

### OHCs

As Web 2.0 technologies are increasingly being used within the health care industry, patients and physicians have begun to participate more actively in health management [22]. Numerous studies have started to explore the benefits of OHCs from different perspectives. For example, Li et al [5] explored the economic success of physicians in the online consultation market. Guo et al [20] analyzed how physicians gained social and economic returns in OHCs. In contrast to traditional medical service delivery, OHCs give patients the opportunity to review the abundant amount of various types of information, and then use this information to choose the physician they wish to consult [23].

**Figure 1:**
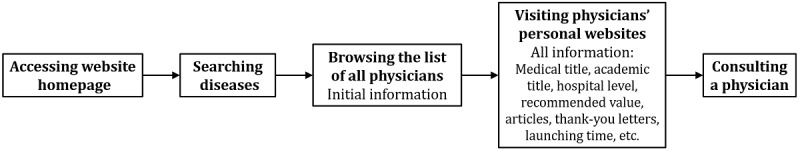
Patient decision-making process in online health communities.

Specifically, the patient decision-making process in OHCs includes several stages and is also a process of gradually selecting physicians based on the information obtained, as shown in Figure 1. According to their diseases, patients can obtain a list of physicians that specialize in that disease and initial information to select alternatives, and then determine whether they should visit the personal website of the physician to get more information. When patients enter the physician’s personal website, they can obtain more comprehensive information (eg, online behaviors, number of patients and visitors, articles) and decide whether or not to consult and how to consult [12]. Thus, a physician’s personal website plays an important role in the patient decision-making process, and the OHC information presented determines whether a patient is only a visitor or can be a customer. Although previous research has explored the effects on patients’ choices of different stages [12,18,24], there is currently no research available that focused on why some physicians are not selected for consultation even if their personal websites are visited by patients.

### Conversion Rate

In electronic commerce (e-commerce), conversion rates represent the proportion of orders to website visitors. For instance, if 100 people visit a website and 3 of them purchase the product, the conversion rate is 3.0% [25]. Prior research has shown that website factors, including website features [26,27] and functionalities [25], and customer factors, including customer reviews and sentiments [28], browsing, and purchasing experiences [29,30], have effects on the conversion rates of websites.

Compared with the conversion rates of other types of websites, research on the conversion rate of a service website is still in a preliminary stage. As service is intangible, inseparable, and heterogeneous [9], Jackson [10] defined the conversion rate of a service website as the proportion of the number of customers to the total number of visitors, and customers were defined as those who successfully locate the information that the service provider wanted them to find (eg, downloadable FAQ, emails the correct support address, or find the answer they were looking for). Cezar and Ögüt [7] analyzed the conversion rates in online hotel booking sites from the reviews of customers, recommendations, and ranking order of the system. Zhou et al [31] investigated the effect of posts generated by other members’ activities in transforming visitors into members in online brand communities. However, there is no research on the conversion rate of OHCs.

Considering the great demand for medical services and limited medical resources [32], the efficiency of an OHC is crucial. Visiting a physician’s personal website is a vital stage in the process of patient decision making, which is the prerequisite for converting visitors to physicians’ personal websites into customers. The number of customers indicates the number of patients a physician has provided medical services for; that is, the number of visitors that have successfully converted. A higher conversion rate of a personal website means that more patients choose this physician for consultation, and this physician can also obtain more social and economic returns [5,20]. The increased conversion rate of all physicians’ personal websites means that the efficiency of the OHC has increased. Therefore, the present study focused on the conversion rate of OHCs; that is, the proportion of customers to the total visitors of a physician’s personal website.

### Multisource Information in OHCs

When customers search for information to make critical purchase decisions, they often use multiple information sources [13-15], including electronic word-of-mouth sources, neutral/third party sources, and manufacturer/retailer sources. Similarly, patients selecting a physician in an OHC also need to search for information from multiple sources. Previous studies have investigated the link between multisource OHC information and patients’ choices. For example, Li el al [18] explored the effects of patients’ rating and physicians’ activeness on the number of patients. Liu et al [16] found that the number of physicians’ appointments was positively associated with their individual offline and online reputations, as well as their organizational offline and online reputations. Yang et al [12] categorized OHC information into system-generated and patient-generated information, and explored their effects on patients’ online searches, evaluations, and decisions.

System-generated information represents pieces of information that are system- or machine-rendered [33]. This online information about products and services from neutral/third parties is considered to be more useful and objective [34], and provides references for customers (eg, ranking orders and recommendations) [35,36]. Matching a customer’s preferences against the most similar customers’ preferences facilitates the purchasing process, as it narrows alternatives down to a few and hence decreases the time spent searching [7]. System-generated information in an OHC is created by the system based on the service provider’s contribution and popularity, which is independent of physicians and patients [12]. Patient-generated information is derived from the patient feedback channel of OHCs and is generated by patients who have experienced a medical consultation (eg, reviews and ratings). Generally, patient-generated information reflects the outcomes of a physician’s service [12], and is related to the perception of the physician’s skill [17] and patients’ choices at different stages [12,18,19].

However, an OHC is an online community that uses information technology to present a medical ecosystem including patients and physicians [4]. OHC information also includes information generated by physicians’ online behaviors and activities [18] (ie, physician-generated information). In an OHC, physicians can provide additional types of consultation than offline hospitals or clinics [37] (eg, network consultation, telephone consultation, appointment registration). In addition, physicians can update personal information, publish articles, reply to consultations, and manage patients in their personal websites. These online behaviors and activities can generate considerable information. Previous studies have shown that the number of articles published by a physician indicates the degree of activeness, knowledge contribution, and professional capital, which affect patients’ choices [18]. Therefore, this study focused on information from three different sources: physician-generated, patient-generated information, and system-generated information.

### Research Model and Hypotheses

The customer decision-making process consists of five stages: needs recognition, information search, pre-evaluation, purchase decision, and postevaluation [38]. This is a process of continually eliminating options relying on multisource information, which is similar to the process of a patient selecting a physician in OHCs. As service is intangible, inseparable, and heterogeneous [9], patients narrow down the alternatives and finally choose one physician to consult by relying on multisource OHC information. Considering the importance of the physician’s personal website for the patient decision-making process, in this study, we focused on the roles of the multisource OHC information (physician-generated information, patient-generated information, and system-generated information) in the process of converting visitors to customers.

#### Physician-Generated Information and Conversion Rate

As the delivery of physicians’ services is based on the platform, the information of physicians’ behaviors and activities is considerable. The more information the physician generates, the more active the physician is on the platform [18]. Conversely, less available physician-generated information represents the behavior of inactive physicians. This kind of information can therefore reveal the delivery process quality of the physician’s service [12]. Patients prefer physicians who are actively involved online and have a high-quality service delivery process [12,18], and physicians’ online behaviors and activities have been shown to affect outpatient visits in hospitals [39]. When patients see a large amount of physician-generated information on a physician’s personal website, they will be more willing to convert as a customer. Therefore, it is hypothesized that *physician-generated information positively affects the conversion rate of a physician’s personal website (H1).*

#### Patient-Generated Information and Conversion Rate

A physician’s personal website also serves as a feedback channel for patients. Feedback from other people who have experienced a certain quality is a useful signal to help receivers understand quality, and is of vital importance in affecting information for receivers’ decision making [40]. Patient-generated information (eg, ratings and reviews) comes from other patients who have consulted the physician. Therefore, a greater amount of patient-generated information in a physician’s personal website indicates a higher number of patients who have selected the physician for consultation. Moreover, patient-generated information reflects a physician’s service outcomes [12]. Such information is more objective and credible than traditional information from friends and family members [41]. Patients prefer to choose a physician who has been consulted by a large number of patients [12] and delivers high-quality service outcomes. Therefore, it is hypothesized that: *Patient-generated information positively affects the conversion rate of a physician’s personal website (H2).*

The coexistence of physician-generated and patient-generated information may cause both items to complement each other in driving patients to choose a consultation after visiting a physician’s personal website. In the e-commerce context, service quality has a significant positive influence on customers’ purchase decisions. Because service is created by the interaction between service provider and receiver, service quality evaluations should concentrate on two dimensions: the service delivery process and service outcomes [42]. Patients require multisource information to judge the quality of the service delivery process and outcomes [12]. Per the preceding discussion, physician-generated information reflects the service delivery process and patient-generated information represents the service outcomes, and the two should complement each other. When patients obtain more physician-generated information or patient-generated information on a physician’s personal website, the possibility of choosing the physician for consultation (ie, the conversion rate of the physician’s personal website) will be enhanced. Therefore, it is hypothesized that: *Physician-generated information and patient-generated information have a complementary relationship in affecting the conversion rate of a physician’s personal website (H3).*

#### System-Generated Information and Conversion Rate

System-generated information is generated by the third-party platform (eg, ranking orders and recommendations) [35,36]. Online product recommendations from third parties are perceived as being more useful in terms of providing accurate information. Many consumers are willing to search for this information to satiate the uncertainty they feel toward information from manufacturers/retailers [34]. The system of an OHC also generates recommendations for physicians. Although this recommendation does not directly reflect a physician’s service quality [12], it is more objective and accurate. In an OHC, patients will form an overall impression through systematic recommendations. Compared with physicians with low recommended value, patients are more willing to assume that physicians who have highly recommended value are credible. There is a reason to believe that the conversion rate of the personal website of a highly recommended physician will be high. Therefore, it is hypothesized that: *System-generated information positively affects the conversion rate of a physician’s personal website (H4).*

#### Moderating Effect of Usage Time

The physician’s personal website is a platform for presenting OHC information, including physician-generated and patient-generated information. Studies have shown that the launching of personal websites by physicians can significantly increase the amount of patient reviews [43] (ie, patient-generated information). The longer the website is used, the more physician-generated and patient-generated information should be presented on the personal website. Moreover, the time the physician uses the personal website also indicates the degree of familiarity with online medical services, and patients prefer to choose a more experienced physician. Therefore, it is hypothesized that: *The usage time of a personal website positively moderates the relationship between physician-generated information and the conversion rate (H5a); The usage time of a personal website positively moderates the relationship between patient-generated information and the conversion rate (H5b).*

**Figure 2:**
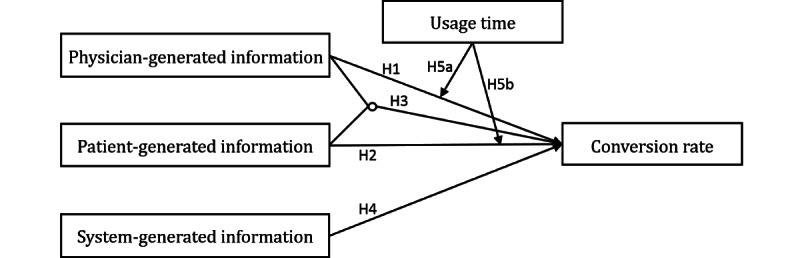
Research model.

The overall research model is schematically shown in Figure 2.

## Methods

**Figure 3:**
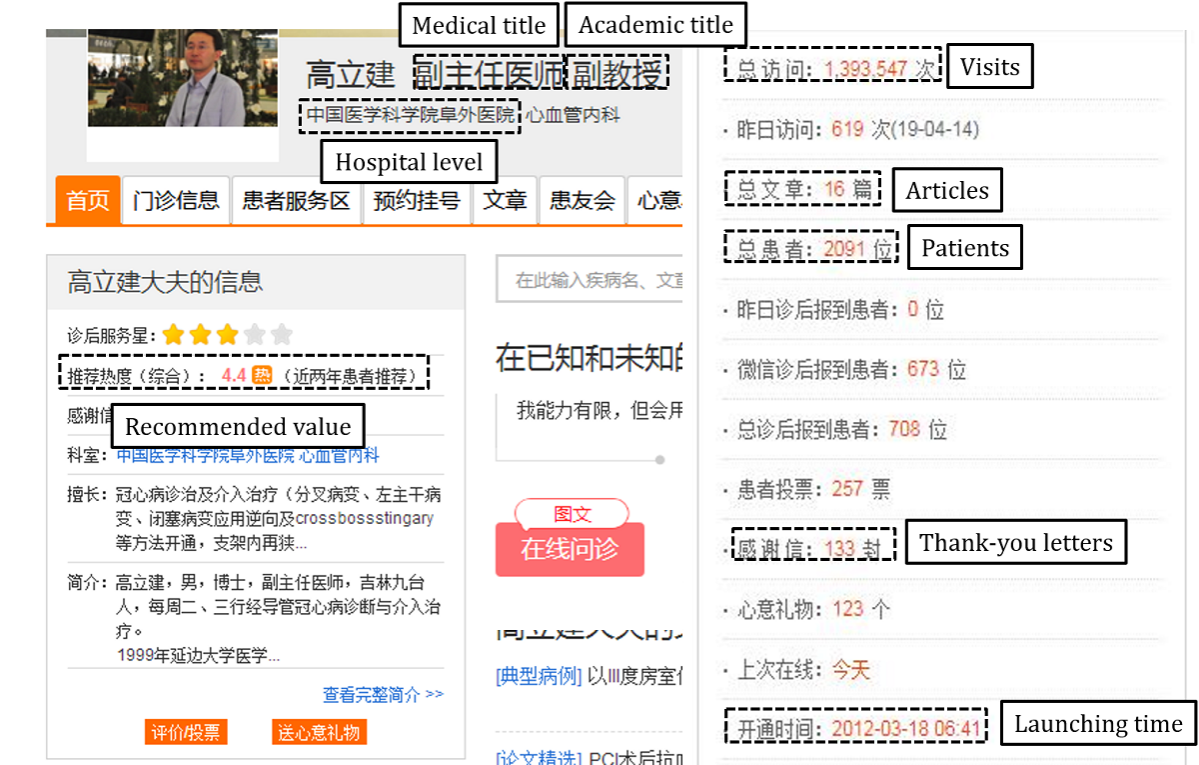
An example of a physician’s personal website.

For this study, we used data from Haodf.com [21], a leading OHC in China, focusing specifically on physicians treating coronary heart disease as an example. A physician’s personal website in Haodf.com presents multisource OHC information, including the number of people who visit that physician’s website, the number of patients who consult that physician, physician-generated information (eg, articles), patient-generated information (eg, thank-you letters), and system-generated information (eg, recommended value). Figure 3 shows an example of a physician’s personal website.

By means of web crawler technology, the data were collected in five time periods: from March 2019 to July 2019 (for 1 month each over these 5 months), covering 2112 physicians. To investigate whether physician-generated, patient-generated, and system-generated information affect the conversion rate of a physician’s personal website, a longitudinal study was designed.

**Figure 4:**
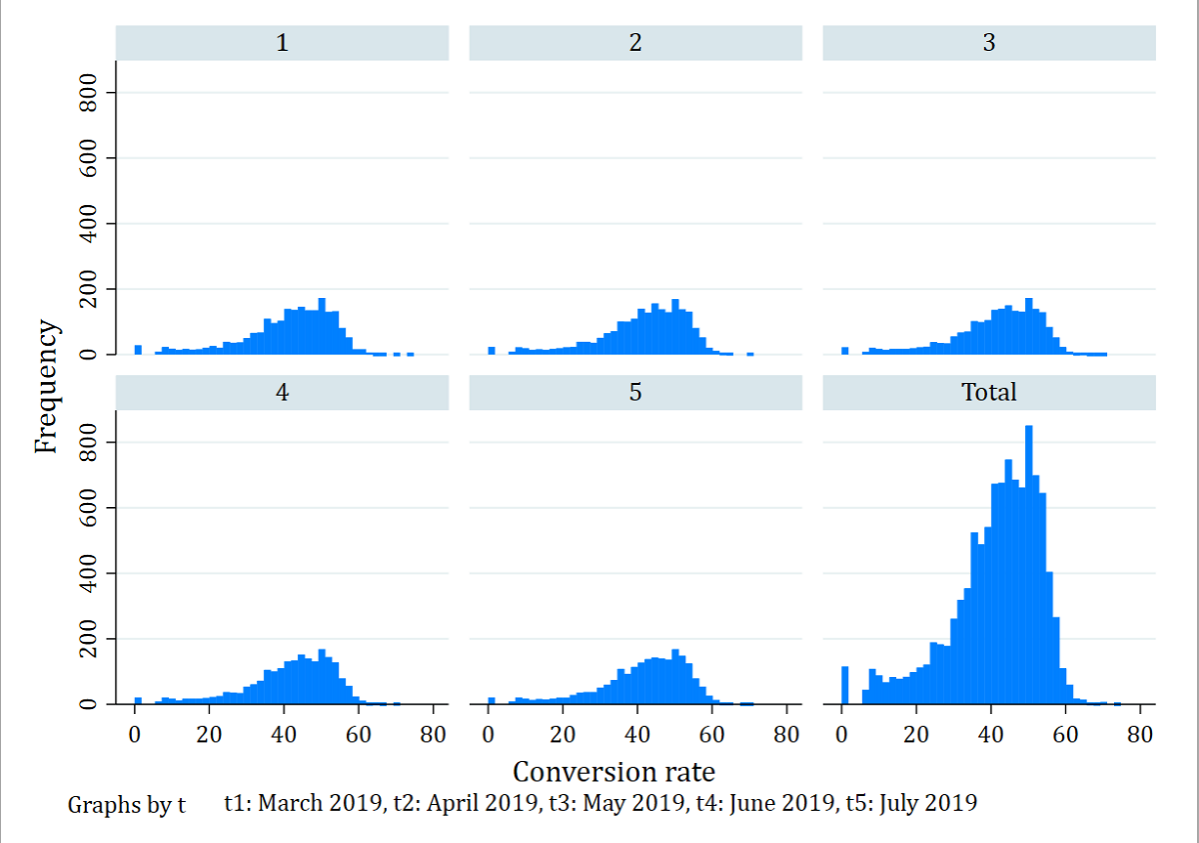
Frequencies of conversion rates in the online health community over five time periods.

Table 1 summarizes the main variables considered in our study. We used the proportion of the number of patients relative to the number of visits before time *t* as a proxy for the conversion rate of a physician’s personal website. The number of patients included those who only consult online, and those who consult again after offline consultation, which can be considered to comprise patients with two kinds of consultations. The mean and maximum number of patients and visits were not an order of magnitude, and their variances were large. Thus, to avoid the calculated conversion rate values being too small, we conducted natural logarithm transformation, ln(*X*+1), as shown in equation (1). Figure 4 shows the frequency statistics of conversion rates over the five time periods, clearly demonstrating variation in conversion rates, which is worth studying.


Conversion rate*_i, t_* (%)=[ln(Patients*_i,t_*+1)/ln(Visits*_i,t_*+ 1)] ×100    **(1)**

The independent variables included physician-generated information, patient-generated information, and system-generated information. Physician-generated information was measured by the number of articles published by the physician before time *t*. Patient-generated information was measured by the number of thank-you letters written by patients after consulting before time *t*. Since the distributions of articles and thank-you letters were nonnormal and some values were 0, natural logarithm transformations were also applied prior to analysis. System-generated information was measured by the recommended value of the system at time *t*, ranging from 1 to 5. The physician’s order in the list is also in accordance with the recommended value. Moreover, usage time was used as a moderator, measured by the difference between the launch time of a physician’s personal website and time *t*.

Control variables included medical title, academic title, and hospital level by the physician at time *t*. These factors represent objective characteristics of a physician, which are reviewed by the system when the physician enters the OHC. A physician’s medical title has four levels: resident physician, attending physician, deputy chief physician, and chief physician, coded from 1 to 4, respectively. The three academic titles for a physician are lecturer, associate professor, and professor, coded from 1 to 3, respectively. Hospitals are also ranked according to three levels coded 1 to 3.

**Table 1:** Variables description.

Variable		Description	Proxy
Conversion rate (dependent variable)		Proportion of customers to visitors who successfully locate information in the physicians’ personal websites and decide to consult.	Patient visits
**Independent variables**			
	Physician-generated information	The information generated by physicians’ behaviors and activities in the OHC^a^.	Articles
	Patient-generated information	The information generated by patients who have experienced medical consultation in the OHC.	Thank-you letters
	System-generated information	The information generated by the OHC system.	Recommended value
Usage time (moderator)		The time since the physician has launched their personal website in the OHC.	Usage time
**Control variables**			
	Medical title	The physician’s administrative position in the hospital, which is a manifestation of the physician’s ranking.	Medical title
	Academic title	The physician’s professional position, which is also a manifestation of the physician’s ranking.	Academic title
	Hospital title	The level of the physical hospital where a physician works.	Hospital level

^a^OHC: online health community.

## Results

### Descriptive Statistics and Correlations

Table 2 and Table 3 show the descriptive statistics and correlations among variables, respectively. The numbers of articles and letters showed a positive correlation with the conversion rate, and the number of articles was positively correlated with the number of letters. The recommended value was positively correlated with the conversion rate. Moreover, the number of articles and letters was each positively correlated with usage time.

**Table 2:** Descriptive statistics (N=10,560).

Variable	Mean (SD)	Minimum	Maximum
Medical title	2.455 (1.463)	0	4
Academic title	0.866 (1.201)	0	3
Hospital level	2.927 (0.378)	0	3
Articles	11.246 (53.232)	0	1315
Thank-you letters	20.738 (66.354)	0	1632
Recommended value	3.816 (0.287)	3.100	5
Usage time	5.058 (3.150)	0	11.420
Patients	529.189 (1390.845)	0	29,613
Visits	439,980 (1302.529)	17	1.81e+07

**Table 3:** Correlations among variables (N=10,560).

Variable		Medical title	Academic title	Hospital level	Ln(Articles+1)	Ln(Thank-you letters+1)	Recommended value	Usage time	Conversion rate
**Medical title**									
	β	1	.583	.008	.029	.099	.138	.185	–.014
	*P* value	—^a^	<.001	.40	.003	<.001	<.001	<.001	.15
**Academic title**									
	β	.583	1	.015	.137	.254	.307	.392	.065
	*P* value	<.001	—	.14	<.001	<.001	<.001	<.001	<.001
**Hospital level**									
	β	.008	.015	1	–.047	.064	.084	.056	–.045
	*P* value	.40	.14	—	<.001	<.001	<.001	<.001	<.001
**ln(Articles+1)**									
	β	.029	.137	–.047	1	.391	.281	.252	.410
	*P* value	.003	<.001	<.001	—	<.001	<.001	<.001	<.001
**ln(Thank-you letters+1)**									
	β	.099	.254	.064	.391	1	.758	.365	.581
	*P* value	<.001	<.001	<.001	<.001	—	<.001	<.001	<.001
**Recommended value**									
	β	.138	.307	.084	.281	.758	1	.292	.431
	*P* value	<.001	<.001	<.001	<.001	<.001	—	<.001	<.001
**Usage time**									
	β	0.185	.392	.056	.252	.365	.292	1	.009
	*P* value	<.001	<.001	<.001	<.001	<.001	<.001	—	.34
**Conversion rate**									
	β	–.014	.065	–.045	0.410	.581	.431	.009	1
	*P* value	.15	<.001	<.001	<.001	<.001	<.001	.34	—

^a^Not applicable.

### Estimation Model

To test the hypotheses, we formulated equation (2). Since the distribution of some variables (*X*) may not be normal and some values were 0, the natural logarithm transformation was used for these variables prior to analysis.


Conversion rate*_i, t_* (%)=[ln(Patients*_i,t_*+1)/ ln(Visits*_i,t_*+ 1)]×100=α_0_ + α_1_Medical title*_i,t_* + α_2_Academic title*_i,t_* + α_3_Hospital level*_i,t_* + α_4_Physician-generated information*_i,t_* + α_5_Patient-generated information*_i,t_* + α_6_System-generated information*_i,t_* + α_7_Physician-generated information*_i,t_* *Patient- generated information*_i,t_* + α_8_Usage time*_i,t_* + α_9_Physician-generated information_i,t_* Usage time*_i,t_* + α_10_Patient-generated information_i,t_* Usage time*_i,t_* + α*_i,t_*      **(2)**

### Regression Results

The results of the Hausman test (χ^2^(11)=238.49, *P*<.001) showed that a fixed-effects model was more suitable for this study. The fixed-effects model assists in controlling for unobserved heterogeneity (ie, heterogeneity among individual physicians) when this heterogeneity is constant over time. Therefore, the results of the fixed-effects model are reported accordingly in the main analysis, as shown in Table 4.

In addition, the results are presented hierarchically. Model 1 contained only control variables, whereas model 2 to model 4 included the independent variables and interaction terms. Model 5 represents the full model that included all of the independent variables as well as the interaction terms.

From model 2, the coefficient for physician-generated information was positive and statistically significant, indicating that physician-generated information positively affects the conversion rate of a physician’s personal website. Therefore, H1 is supported. The results of model 2 also showed that the coefficient of patient-generated information was positive and statistically significant, suggesting that patient-generated information positively affects the conversion rate of a physician’s personal website. Therefore, H2 is supported.

Model 3 showed that the interaction between physician-generated information and patient-generated information was negative and significant. This means that the effect of physician-generated and patient-generated information on the conversion rate is a substitute relationship rather than a complementary relationship, which is in contrast to H3.

Model 2 showed that the coefficient of system-generated information was positive and statistically significant, suggesting that system-generated information positively affects the conversion rate of a physician’s personal website. Therefore, H4 is supported.

**Figure 5:**
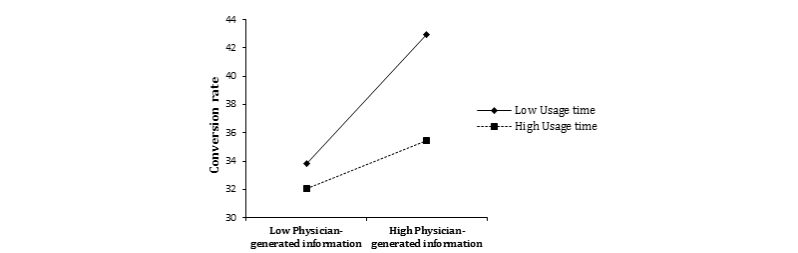
The moderating effect of usage time on physician-generated information and the conversion rate.

Model 4 showed that the interaction between physician-generated information and usage time was negative and significant. This finding suggests that the relationship between physician-generated information and the conversion rate can be negatively moderated by the usage time of a personal website. That is, when the usage time of a personal website is lower, the relationship between physician-generated information and the conversion rate is stronger, as shown in Figure 5. Therefore, H5a is not supported.

**Figure 6:**
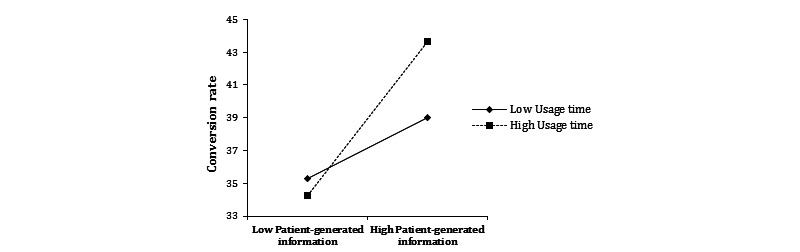
The moderating effect of usage time on patient-generated information and the conversion rate.

By contrast, model 4 also showed that the interaction between patient-generated information and usage time was positive and significant. This finding suggests that the effect of patient-generated information on the conversion rate is stronger for physicians with high usage time of their personal websites, as shown in Figure 6. Therefore, H5b is supported.

**Table 4:** Regression results (fixed-effects models).

Variable	Model 1^a^		Model 2^b^			Model 3^c^		Model 4^d^		Model 5^e^	
	β^a^ (SE)	*P* value	β (SE)	*P* value	β (SE)		*P* value	β (SE)	*P* value	β (SE)	*P* value
Constant	41.206 (1.300)	<.001	21.759 (1.867)	<.001	33.068 (1.251)		<.001	35.119 (1.427)	<.001	21.156 (2.052)	<.001
Medical title	–.048 (.014)	.001	.006 (.014)	.68	.015 (.014)		.27	.010 (.017)	.52	.007 (.016)	.64
Academic title	.012 (.028)	.66	–.046 (.026)	.08	–.050 (.026)		.06	–.032 (.026)	.23	–.026 (.026)	.33
Hospital ranking	.023 (.444)	.96	–.046 (.420)	.91	.025 (.417)		.95	.064 (.419)	.88	.010 (.413)	.98
PHI^f^	N/A^g^	N/A	3.009 (.127)	<.001	4.436 (.165)		<.001	3.993 (.167)	<.001	5.070 (.185)	<.001
PI^h^	N/A	N/A	1.694 (.131)	<.001	2.843 (.145)		<.001	.757 (.196)	<.001	1.315 (.202)	<.001
SI^i^	N/A	N/A	3.336 (.381)	<.001	N/A		N/A	N/A	N/A	3.465 (.382)	<.001
PHI×PI	N/A	N/A	N/A	N/A	–.904 (.067)		<.001	N/A	N/A	–.970 (.071)	<.001
Time^j^	N/A	N/A	N/A	N/A	N/A		N/A	–.346 (.160)	.03	–.429 (.160)	.008
PHI×Time	N/A	N/A	N/A	N/A	N/A		N/A	–.333 (.040)	<.001	–.147 (.041)	<.001
PI×Time	N/A	N/A	N/A	N/A	N/A		N/A	.325 (.041)	<.001	.382 (.041)	<.001

^a^R^2^ within=0.003; R^2^ (between)=0.002; R^2^ (overall)=0.001.

^b^R^2^ (within)=0.110; R^2^ (between)=0.331; R^2^ (overall)=0.329.

^c^R^2^ (within)=0.121; R^2^ (between)=0.334; R^2^ (overall)=0.332.

^d^R^2^ (within)=0.113; R^2^ (between)=0.355; R^2^ (overall)=0.353.

^e^R^2^ (within)=0.139; R^2^ (between)=0.344; R^2^ (overall)=0.343.

^f^PHI: Physician-generated information.

^g^N/A: not applicable.

^h^PI: Patient-generated information.

^i^SI: System-generated information.

^j^Time: Usage time.

### Robustness Check

To check the robustness of the results, we considered the time effect and used the two-way fixed-effects model to rerun the estimation model (Equation 2). In this case, time was defined as a dummy variable and *t*1 (March 2019) was used as the baseline period. The new estimation model is shown in equation (3), and the results are presented in Table 5, which were consistent with the results of the previous model (Table 4). In addition, the joint significance of the time dummy variable was tested, which confirmed that the time effect should be included in the new estimation model. The robustness check results suggested that H1, H2, H4 and H5b were supported.


Conversion rate*_i, t_* (%)=[ln(Patients*_i,t_*+ 1)/ln(Visits*_i,t_*+1)]×100
=β_0_ + β_1_Medical title*_i,t_* +β_2_Academic title*_i,t_* + β_3_Hospital level*_i,t_* + β_4_Physician-generated information*_i,t_* + β_5_Patient-generated information*_i,t_* + β_6_System-generated information*_i,t_* + β_7_Physician-generated information_i,t_* Patient-generated information*_i,t_* + β_8_Usage time*_i,t_* + β_9_Physician-generated information_i,t_*Usage time*_i,t_* + β_10_Patient-generated information_i,t_*Usage time*_i,t_* + β_11_*t2* + β_12_*t*3 + β_13_*t4* + β_14_*t5* + β*_i,t_*      **(3)**

**Table 5:** Robustness check (fixed-effects models).

Variable	Model 1^a^		Model 2^b^		Model 3^c^			Model 4^d^		Model 5^e^	
	β (SE)	*P* value	β (SE)	*P* value		β (SE)	*P* value	β (SE)	*P* value	β (SE)	*P* value
Constant	40.801 (1.301)	<.001	21.470 (1.883)	<.001		33.180 (1.255)	<.001	158.795 (9.053)	<.001	141.657 (9.053)	<.001
Medical title	.006 (.014)	.89	–.013 (.014)	.73		–.021 (.038)	.58	–.044 (.038)	.25	–.038 (.038)	.31
Academic title	–.001 (.029)	.98	–.041 (.028)	.14		–.040 (.027)	.14	–.009 (.027)	.75	–.005 (.027)	.85
Hospital rank	.060 (.443)	.89	–.038 (.420)	.93		.029 (.417)	.95	.074 (.414)	.86	.020 (.409)	.96
PHI^f^	N/A^g^	N/A	2.986 (.128)	<.001		4.443 (.166)	<.001	4.034 (.165)	<.001	5.081 (.183)	<.001
PI^h^	N/A	N/A	1.637 (.135)	<.001		2.823 (.148)	<.001	.628 (.194)	.001	1.168 (.201)	<.001
SI^i^	N/A	N/A	3.446 (.387)	<.001		N/A	N/A	N/A	N/A	3.446 (.378)	<.001
PHI*PI	N/A	N/A	N/A	N/A		–.905 (.067)	<.001	N/A	N/A	–.945 (.070)	<.001
Time^j^	N/A	N/A	N/A	N/A		N/A	N/A	–25.554 (1.831)	<.001	–24.984 (1.805)	<.001
PHI×Time	N/A	N/A	N/A	N/A		N/A	N/A	–.345 (.039)	<.001	–.164 (.041)	<.001
PI×Time	N/A	N/A	N/A	N/A		N/A	N/A	.335 (.041)	<.001	.391 (.041)	<.001
t2^k^	.121 (.039)	.002	.035 (.037)	.34		.015 (.037)	.67	1.944 (.144)	<.001	1.894 (.142)	<.001
t3^l^	.197 (.039)	<.001	.057 (.037)	.12		.031 (.037)	.40	4.108 (.298)	<.001	3.997 (.293)	<.001
t4^m^	.254 (.039)	<.001	.072 (.038)	.06		.031 (.037)	.40	6.386 (.462)	<.001	6.222 (.456)	<.001
t5^n^	.307 (.117)	.009	–.011 (.112)	.92		–.089 (.111)	.42	8.299 (.612)	<.001	8.108 (.604)	<.001

^a^R^2^ within=0.009; R^2^ (between)=0.003; R^2^ (overall)=0.000.

^b^R^2^ (within)=0.110; R^2^ (between)=0.330; R^2^ (overall)=0.328.

^c^R^2^ (within)=0.121; R^2^ (between)=0.334; R^2^ (overall)=0.332.

^d^R^2^ (within)=0.132; R^2^ (between)=0.001; R^2^ (overall)=0.001.

^e^R^2^ (within)=0.157; R^2^ (between)=0.001; R^2^ (overall)=0.001.

^f^ PHI: Physician-generated information.

^g^N/A: not applicable.

^h^PI: Patient-generated information.

^i^SI: System-generated information.

^j^Time: Usage time.

^k^*t*2: April 2019.

^l^*t*3: May 2019.

^m^*t*4: June 2019.

^n^*t*5: July 2019.

## Discussion

### Principal Findings

The aim of this study was to explore the conversion rate of OHCs. Considering the importance of the physician’s personal website in the patient decision-making process, the conversion rate in this study was considered to be the proportion of customers among visitors of a physician’s personal website. The results showed that physician-generated information had a positive effect on the conversion rate. Corresponding to previous research, the number of articles published by a physician reflects their activeness and affects patients’ choices [18], thereby affecting social and economic returns [20].

Just as reviews and posts generated by customers or members affect the conversion rate of websites [7,28,31], patient-generated information also positively affects the conversion rate of physicians’ personal websites. Physician-generated and patient-generated information serve as two clues to reflect two dimensions of a physician’s service quality: the service delivery process and service outcomes. The results showed that there was no complementary relationship but rather a substitute relationship between physician-generated and patient-generated information. A possible explanation for this finding is that both physician-generated and patient-generated information contain information about a physician’s quality, and these two types of online quality information may have some overlap. More patient-generated information also means that there are many patients who have chosen to consult this physician. In general, patients believe that a good physician with more patients should be very busy and should not have sufficient time to contribute much online. As a result, patients may tend to believe that the signals are manipulated by the physician or the system [5].

This study also found that system-generated information positively affected the conversion rate of a physician’s personal website. This result is consistent with a prior study suggesting that ranking orders and recommendations generated by the system affect the conversion rates of online hotel booking websites [7].

Although physician-generated and patient-generated information on a physician’s personal website are cumulative since the launch of the website, the moderating effects of usage time on the two types of information differed. That is, the usage time of a personal website had a positive moderating effect on the relationship between patient-generated information and the conversion rate, but had a negative moderating effect on the relationship between physician-generated information and the conversion rate. This may reflect that patient-generated information grows in proportion to the time a physician uses the personal website, whereas physician-generated information grows more slowly. It may also be related to the fact that compared with physician-generated information, patient-generated information is more relevant to the physician’s familiarity with online medical services.

### Theoretical Implications

This study offers theoretical contributions in the following ways. First, conversion rates of other types of websites (ie, retail or e-commerce websites, potential customer generation websites, and content websites) and their influencing factors have been extensively studied. This study focused on the conversion rate of a service website (OHC). The conversion rate in this study was considered as the proportion of customers among patients who visited a physician’s personal website. Paying attention to the conversion rate of physicians’ personal websites and analyzing its influencing factors can provide clearer understanding of the patient decision-making process in OHCs.

Second, previous studies divided OHC information into patient-generated information and system-generated information according to the source. By contrast, this study also added the component of physician-generated information and thus extended research on multisource OHC information. The results showed that multisource OHC information (physician-generated, patient-generated, and system-generated information) positively affected the conversion rate of a physician’s personal website.

Third, this study employed usage time as OHC information related to the familiarity of physicians with online medical services, which was analyzed together with physician-generated and patient-generated information. The results demonstrated that the moderating effects of usage time on the two kinds of OHC information were different. From this perspective, this study extends the understanding of OHC information from the time dimension.

### Implications for Practice

This study provides several relevant practical implications. First, for the manager, analyzing and improving the conversion rate of a physician’s personal website is conducive to improving the efficiency of the entire platform. Our results further indicate that system-generated information (eg, recommended value) positively affects the conversion rate of a physician’s personal website. The website manager should improve the accuracy of system-generated information and update it in time.

Second, the results showed that physician-generated information affected the conversion rate of a physician’s personal website. Therefore, physicians who want to improve the conversion rate of their websites must be active and hardworking, and pay attention to the information left by their own behaviors and activities.

Third, although both physician-generated and patient-generated information positively affected the conversion rate, they showed a substitute relationship rather than a complementary relationship. In other words, these two types of OHC information have distinct roles in affecting the conversion rate of a physician’s personal website. Physicians can mainly focus on either physician-generated information or patient-generated information, taking into account their specific objectives or resource constraints in different situations. Additionally, physicians should distinguish between the service delivery process and service outcomes in patients’ assessments of service quality.

Fourth, the results showed that physician-generated and patient-generated information had a time effect on the conversion rate, and the two were different. Therefore, physicians should not only pay attention to the cumulative amount of physician-generated and patient-generated information in personal websites but also the directly proportional effects to their usage time.

### Limitations and Future Research

This study has certain limitations. First, the interpretation of the findings is limited by using data from only one Chinese OHC, Haodf.com, and one type of physician, specialists in coronary heart disease. Therefore, collecting data from physicians with various types of expertise on different platforms simultaneously is necessary to further verify the research model. Second, it is difficult to distinguish between two types of consultations: patients who browse and consult online, and patients who browse online but consult offline. In this study, we used the number of patients and visits collected by the platform, which might have resulted in the loss of some information. Third, it is difficult to obtain patient-level data from this platform, since the platform tends to mask the users’ names to protect patient privacy. Therefore, we were not able to empirically examine how different patients make choices based on available OHC information. Consequently, this exclusion may be a potential limitation of this study.

### Conclusions

In this study, the conversion rate of OHCs was analyzed (ie, how to convert visitors into customers). We hypothesized that multisource OHC information (physician-generated, patient-generated, and system-generated information) would affect the conversion rate, and that usage time would moderate the relationships between physician-generated information, patient-generated information, and the conversion rate. Short-term panel data over five time periods (months) were used to test these hypotheses. The results indicate that physician-generated, patient-generated, and system-generated information positively affect the conversion rate. In addition, physician-generated and patient-generated information have a substitute relationship rather than a complementary relationship in affecting the conversion rate. Moreover, the usage time of a personal website positively moderates patient-generated information, but negatively moderates physician-generated information.

## Abbreviations

e-commerce: electronic commerce
OHC: online health community
